# Agonist efficiency links binding and gating in a nicotinic receptor

**DOI:** 10.7554/eLife.86496

**Published:** 2023-07-03

**Authors:** Dinesh C Indurthi, Anthony Auerbach

**Affiliations:** 1 Department of Physiology and Biophysics, University at Buffalo, State University of New York Buffalo United States; Semmelweis University Hungary; National Institute of Neurological Disorders and Stroke, National Institutes of Health United States

**Keywords:** acetylcholine, allostery, induced fit, receptor theory, dose-response, efficacy, affinity, Other

## Abstract

Receptors signal by switching between resting (C) and active (O) shapes (‘gating’) under the influence of agonists. The receptor’s maximum response depends on the difference in agonist binding energy, O minus C. In nicotinic receptors, efficiency (η) represents the fraction of agonist binding energy applied to a local rearrangement (an induced fit) that initiates gating. In this receptor, free energy changes in gating and binding can be interchanged by the conversion factor η. Efficiencies estimated from concentration-response curves (23 agonists, 53 mutations) sort into five discrete classes (%): 0.56 (17), 0.51(32), 0.45(13), 0.41(26), and 0.31(12), implying that there are 5 C versus O binding site structural pairs. Within each class efficacy and affinity are corelated linearly, but multiple classes hide this relationship. η unites agonist binding with receptor gating and calibrates one link in a chain of coupled domain rearrangements that comprises the allosteric transition of the protein.

## Introduction

The primary job of a receptor is to convert chemical energy from ligand binding into mechanical work of protein conformational change. Each agonist has two affinities that measure how strongly it binds to resting and active target sites, and an efficacy that measures how well it activates once bound (Cecchini and Changeux, 2022; Ehlert, 2015; Foreman et al., 2011). A third agonist property, efficiency (η; eta), is the correlation between efficacy and affinity and is the receptor’s output/input energy ratio (Nayak et al., 2019). Here, we describe and interpret the distribution of η values estimated from concentration(dose)-response curves (CRCs) of adult-type skeletal muscle nicotinic acetylcholine receptors (AChRs) activated by different agonists and having different binding site mutations.

AChRs are five subunit, ligand-gated ion channels that have two neurotransmitter sites in the extracellular domain (ECD), and a narrow equatorial gate in the lumen of the transmembrane domain (TMD) that governs the passive flow of cations across the membrane (Gharpure et al., 2020; Unwin, 1995; Zarkadas et al., 2022). AChRs are allosteric proteins that alternate spontaneously between global off (C, closed-channel) and on (O, open-channel) conformations in which each neurotransmitter site binds agonists weakly (with low affinity; LA) or strongly (with high affinity; HA) (Cecchini and Changeux, 2022; Jackson, 2006; Phillips, 2020). The AChR neurotransmitter sites and gate are separated by ~60 Å, but both regions change structure and function within the gating isomerization, C_LA_⇄O_HA_.

**Figure 1.**
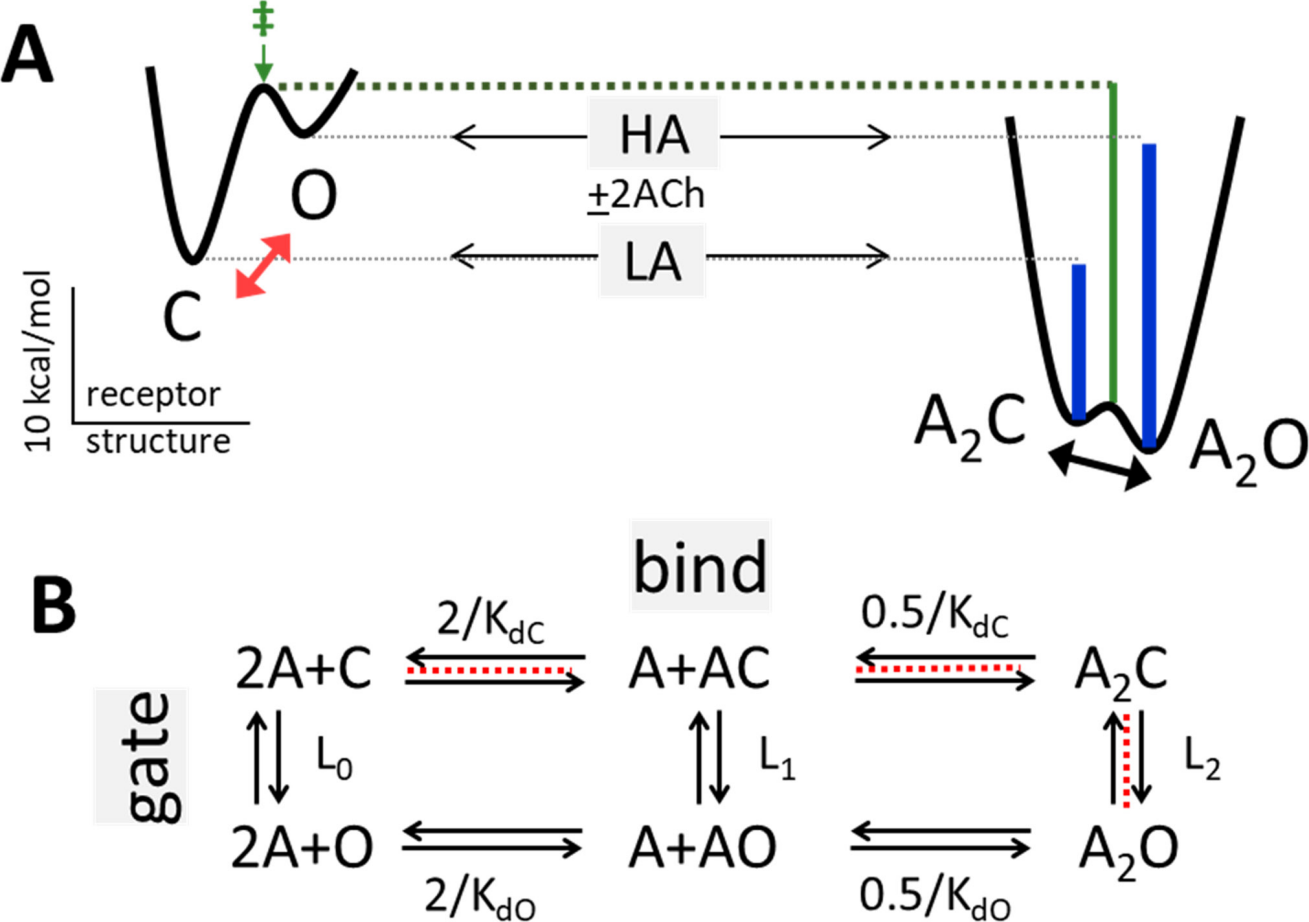
Bind and gate. (**A**) Potential energy surfaces (landscapes) for receptor activation without (left) and with (right) bound agonists (**A**). C, closed channel and low affinity (LA); O, open channel and high affinity (HA). Red, without agonists intrinsic C→O gating is uphill; blue lines, agonists increase P_O_ because they bind more favorably to O; green, agonists also stabilize the gating transition state (‡) to increase the opening rate constant because the LA→HA induced fit (‘hold’, Figure 2) occurs at the start of the gating conformational change. Calibration: ACh, adult-type AChRs, –100 mV, 23 °C. (**B**) Corresponding reaction scheme (2 equivalent and independent neurotransmitter sites). Horizontal, ligand-protein complex formation; K_dC_ and K_dO_, LA and HA equilibrium dissociation constants to C and O. Vertical, global conformational change; L_n_, gating equilibrium constant with n bound agonists. Red dashed line, pathway that determines the CRC when L_0_ is negligible (Equation 4a, Equation 4b, Equation 4c, Equation 4d, Methods).

In adult-type AChRs and without any bound agonists, the probability of the O conformation (P_O_) is small (~10^–6^) so baseline current is negligible (Jackson, 1986; Nayak et al., 2012; Purohit and Auerbach, 2009). However, when both agonist sites are occupied by the neurotransmitter ACh, the increase in favorable binding free energy realized in the spontaneous isomerization from C to O increases P_O_ substantially (to ~0.96), to generate a cellular current (Figure 1).

Our approach to investigating the AChR allosteric transition is to measure free energy changes associated with each component event. Energy and structure are related, and a longer term goal is to associate these energy changes with those in structure. Briefly, we use electrophysiology to compile a CRC and from the minimum, maximum and midpoint calculate the ligand’s LA and HA equilibrium dissociation constants for binding to C and to O (K_dC_ and K_dO_). The logs of these are proportional to the LA and HA binding free energies, ΔG_LA_ and ΔG_HA_. As shown below, agonist efficiency depends only on these two values.

Previously, it was observed that in AChRs the ratio ΔG_LA_/ΔG_HA_ is approximately the same for a group of agonists despite wide variation in individual affinities and efficacies (Jadey and Auerbach, 2012). Within this group, whose members included full agonists (like ACh), partial agonists (like nicotine) and extremely weak agonists (like choline), the binding free energy ratio was 0.51±0.01 (mean ± sd). That is, for all members in the group binding to O is ~two-fold stronger than to C, regardless of agonist affinity or efficacy. Additional experiments revealed two more groups having a shared binding energy ratio, either 0.58 (for example, epibatidine) or 0.46 (for example, tetramethylammonium) (Indurthi and Auerbach, 2021; Nayak et al., 2019). In these groups, agonist binding free energy increases by ~1.7 and ~2.2-fold in the channel-opening conformational change.

The observation of a common ΔG_LA_/ΔG_HA_ ratio for even one group of ligands is remarkable because it implies that the only 2 events in receptor activation that involve the agonist directly - LA binding to C and the switch to HA that happens within gating - are not independent. Rather, the constant ratio indicates that the energy (structure) changes that underpin LA binding to C and HA binding to O are related (Auerbach, 2016; Jadey and Auerbach, 2012; Purohit et al., 2014).

Below, we define η empirically as the receptor’s output/input energy ratio, and propose that in AChRs it is the fraction of agonist binding energy applied to the mechanical work of a local rearrangement of the neurotransmitter site (an induced fit) that initiates the gating conformational cascade (Nayak et al., 2019). In principle, η can be calculated from any CRC to estimate the degree of coupling between the maxima of receptor output (efficacy) and agonist input (affinity).

We measured η for various agonists of AChRs, both wild-type (wt) and following mutation of a binding site residue. Here we report 16 new values (shown in Tables 1 and 2) that, combined with 60 previous measurement describe a spectrum of 5 η classes. The presence of multiple η classes obscures the underlying correlations between affinity and efficacy and, further, suggests that there are multiple C versus O binding site structures. Importantly, the existence of efficiency classes highlights that binding and gating are energy-linked stages of a unified allosteric transition.

**Table 1.** Agonist efficiencies. Table 1—source data 1.Agonist efficiency.

Agonist	EC_50_ (μM) (sem)	P_O_^max^ (sem)	K_dC_ (μM)	K_dO_ (nM)	c	η	n
BzTMA^a^	1070 (200)	0.60 (0.05)	930 (112.0)	650 (90)	1424 (18.0)	0.51 (0.01)	3
Dec^b^	190 (20)	0.79 (0.03)	90 (7.0)	140 (10)	643 (21)	0.41 (0.01)	4
SCh^a^	20 (3)	0.84 (0.02)	50 (4.0)	20 (1)	3177 (118)	0.45 (0.01)	3
BzTEA^b+c^	2 (0.1)	0.85 (0.02)	0.80 (0.3)	3 (2)	316 (14)	0.29 (0.00)	3
TriMAa^b^	16000 (1200)	0.78 (0.03)	7720 (316.0)	12580 (670)	615 (18)	0.57 (0.01)	4

Mean EC_50_ and P_o_^max^ were measured from each CRC (intra-cluster interval duration histograms in Figure 3 and Figure 3—figure supplement 1), with standard error of mean (sem). K_dC_ and K_dO_ were calculated (Equation 4a) after correcting the background mutations that only changed L_0_. c, coupling constant (K_dC_/K_dO_); η, efficiency (Equation 2); error estimates (calculated by error propagation) for calculated K_dC_, K_dO_, c, and η values given by (sem); n, number of CRCs. Membrane potential, +70 mV (to minimize channel block by the agonist). Agonist structures are in Figure 5; superscripts indicate mutation backgrounds: ^a^εS450W, ^b^εL269F, ^c^εE181W that increase L_o_ (increase responses to weak agonists).

## Results

### Background and definitions

**Figure 2.**
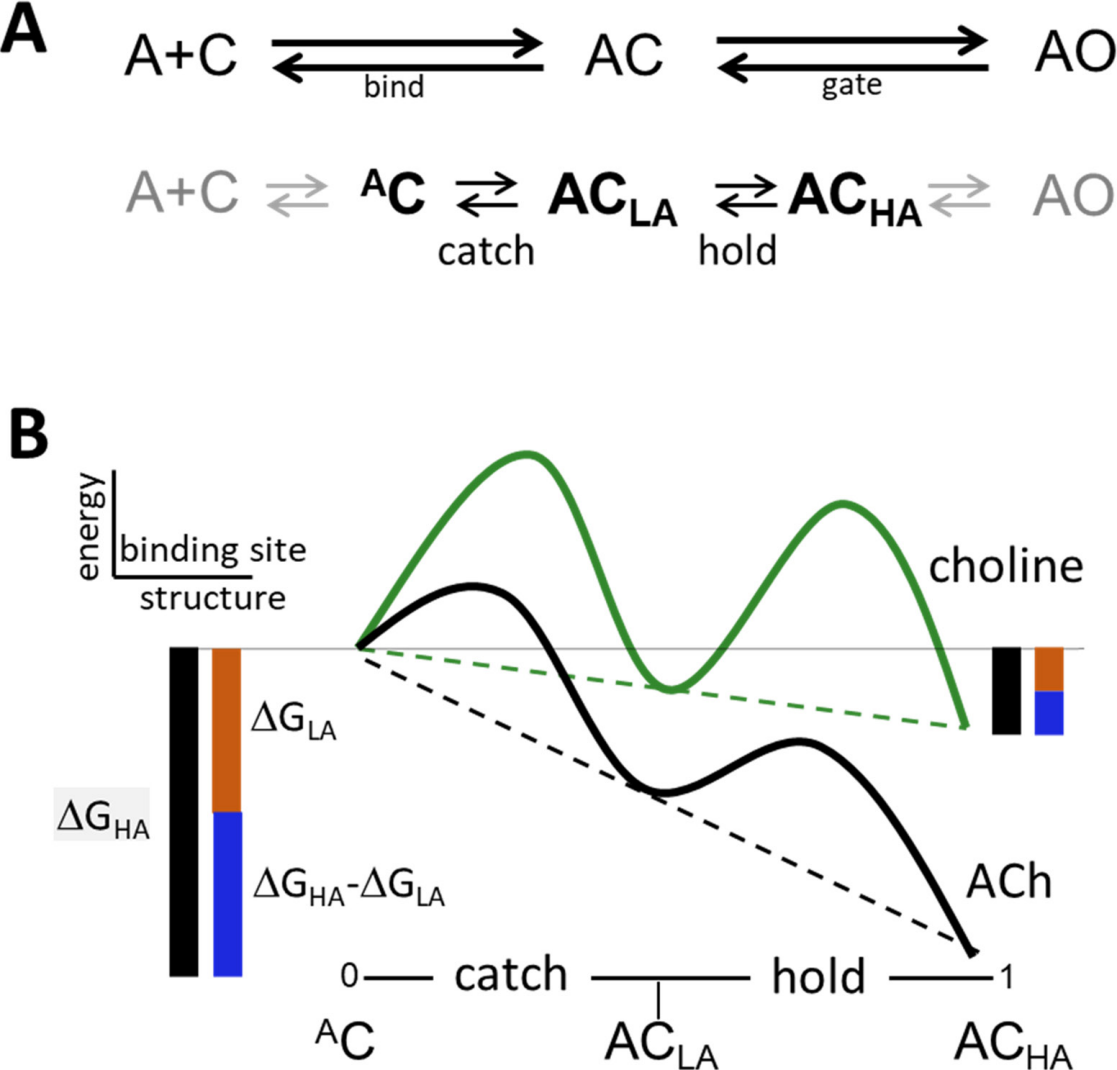
Catch and hold. (**A**) Bind and gate. Top, as one-step reactions. Bottom, as composite reactions. The main undetected intermediate states are ^A^C, the encounter complex (inside bind) and AC_HA_, a high affinity and closed channel state (inside gate). Black, the 2 agonist-dependent induced fit rearrangements are called catch (^A^C⇄AC_LA_) and hold (AC_LA_⇄AC_HA_). Gray, diffusion (A+C⇄^A^C) and receptor conformation changes in other domains (AC_HA_⇄AO) are approximately agonist-independent (see Figure 2—figure supplement 1). (**B**) Catch-and-hold free energy landscape. An η class indicates that catch and hold are linked in a linear free energy relationship (LFER; dashed lines) (Howard, 2001). Green, weak agonist; black, strong agonist. Ligand binding energy ‘tilts’ the entire landscape to the total extent ΔG_HA_ (black side bars). For relative agonist actions, the energy change in catch (brown) determines K_dC_ and the energy change in hold (blue) determines the coupling constant. η is the fraction of the total applied to hold (blue/black) and η is the fraction apllied to catch (brown/black). Agonist affinity and efficacy differ substantially, but η is constant and depends only on the left-right position of AC_LA_ in the reaction.

The standard conception of receptor activation incorporates two seemingly disparate events – bind, the formation of a ligand-protein complex, and gate, the global isomerization of the protein (Figure 1). However, as described below (Figure 2), in AChRs these are composite reactions and are connected by a pair of local, induced-fit rearrangements of the agonist site (Jadey and Auerbach, 2012).

For clarity, we define the universal agonist attributes affinity and efficacy. Affinity is the strength at which the ligand binds to its target site. In receptors ligands have two affinities, weak binding to C and strong binding to O (Figure 1). The corresponding binding free energies, ΔG_LA_ and ΔG_HA_, are calculated (in kcal/mol) as +RT times the natural logarithms of the apparent K_dC_ and K_dO_, where R is the gas constant and T is the absolute temperature (RT = 0.59 at 23 °C). For ACh at adult-type binding sites K_dC_ = 174 μM (ΔG_LA_ = −5.1 kcal/mol) and K_dO_ = 29 nM (ΔG_HA_ = −10.2 kcal/mol) (Jadey and Auerbach, 2012).

Efficacy can be defined in several ways. Oftern, it is simply the high-concentration asymptote (maximum response) of an unnormalized CRC that in our experiments is P_O_^max^. Considering just bind and gate (Figure 1B), this limit depends only on the fully-liganded gating equilibrium constant L_2_ so this constant, too, defines agonist efficacy (Equation 4a). Another definition derives from considering the full cycle of receptor activation. In adult-type AChRs the 2 neurotransmitter binding sites are approximately equivalent and independent and there is no significant input of external energy (Nayak and Auerbach, 2017), so

(1) $$\frac{L_{2}}{L_{0}}={\left(\frac{K_{dC}}{K_{dO}}\right)}^{2}.$$

The subscripts of the gating equilibrium constants (L) refer to the number of bound agonists, and the equilibrium dissociation constant (K_d_) ratio is called the coupling constant (c). L_0_ is agonist-independent so differences in L_2_ (efficacy) among agonists depend only on differences in c. Below, we use the logarithm of c (λ) as the index of relative agonist efficacy,

$$\lambda =\Delta G_{HA}-\Delta G_{LA}.$$

The relative efficacy of an agonist depends only on the difference between binding free energies, O minus C (blue lines in Figure 1A).

Unlike voltage and mechanical stimuli, a small, thermalized ligand can deliver only a small force to a large receptor (Howard, 2001). The tiny momentum imparted to the protein by the ligand is obscured by those from collisions with water molecules. In the absence of external energy, agonists promote conformational change only by providing more favorable (stabilizing) binding energy to active compared to resting states that interconvert spontaneously. This mechanism likely pertains to all large receptors activated by small agonists.

Efficiency (η) is defined empirically as the maximum output/input energy ratio (the efficacy/high-affinity energy ratio),

(2) $$\begin{array}{l} \eta =\frac{\left(\Delta G_{HA}-\Delta G_{LA}\right)}{\Delta G_{HA}}\\ \;\;\;=1-\frac{\Delta G_{LA}}{\Delta G_{HA}} \end{array}$$

Accordingly, the agonist-dependent free energy changes in gating (ΔG_HA_-ΔG_LA_) and in binding (ΔG_HA_) are interchangeable, with η as the conversion factor. Again, efficacy (λ) is a free energy *difference* and the maximum amount the agonist can deliver to the receptor’s gating machinery, and efficiency (η) is a free energy *ratio* and this amount normalized by the ligand’s maximum binding energy. With regard to equilibrium dissociation constants, λ relates to log(K_dC_/K_dO_) and η relates to log(K_dC_)/log(K_dO_) (Figure 7—figure supplement 2).

Insofar as K_dC_ and K_dO_ are universal agonist attributes, so too is η. We propse that an η value can be calculated from the ratio of logarithms of the 2 dissociation constants for every agonist of every receptor. However, this does not imply that η has a physical meaning, or that all agonists of a given receptor have the same (or a unique) η value. Equation 2 only shows how to calculate η from the 2 equilibrium dissociation constants. We estimated these from CRCs, but other experimental approaches would suffice.

In AChRs η does have a physiochemical meaning because K_dC_ and K_dO_ derive mainly from a pair of induced fit rearrangements at the ligand site. Although bind and gate are usually denoted as single-step events (Figure 1B), in AChRs both are composite reactions that harbor intermediate states that are too short-lived to be detected individually and directly (Figure 2, Figure 2—figure supplement 1). Induced fits are common, and undetected intermediate states have been invoked with great success previosuly to explain experimental results (for example ES in enzymology, and AC in pharmacology). Here, we invoke intermediate states to deconstruct η (Figure 2).

**Figure 2—figure supplement 1.**
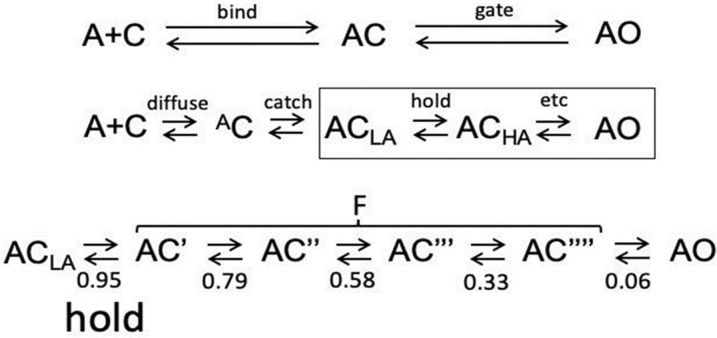
Inside bind and gate. See Figure 1B for 2 equivalent sites. LA, low affinity; HA, high affinity. (**Top**). Standard activation scheme; bind and gate transitions bracket AC (a LA closed state, Delcastillo, et al., 1957). In gate, both channel conductance and binding site affinity increase (AC_LA_⇄AO_HA_). **Middle**. Expanded activation scheme showing undetected intermediates inside bind and gate. In bind, the agonist arrives at the target by diffusion (*diffuse*) and forms an ultra-LA ^A^C encounter complex, followed by the first stage of the induced fit (*catch^1^*) that forms AC_LA_. In gate (boxed), the second stage of the rearrangement (*hold*) forms AC_HA_, followed by additional rearrangements in distant domains (*etc*) that eventually lead to AO_HA_. Experimental K_dC_ and K_dO_ values are dominated by energy changes in the two stages of the induced fit. **Bottom**. Expansion of gating. The distribution of Φ values^2^ suggests that the global isomerization involves passage through 4 short-lived (~100 ns) C_HA_ states (denoted with ‘) associated with sequential rearrangements of the ECD (*twist*), TMD (*tilt*) and gate region (*dilation*), followed by pore water/membrane movements to allow ion transit (*pop*); Φ value given below each transition (Purohit et al., 2013; Gupta et al., 2017). The only agonist dependent rearrangements are catch (^A^C⇄AC_LA_; ΔG_LA_) and hold (AC_LA_⇄AC’; DG_HA_-ΔG_LA_), stages of the induced fit linked in a LFER (Figure 2). Together, sojourns in the ensemble of C_HA_ states appear in single-channel currents as a brief gap (F for *‘flip’*) (Auerbach, 1993; Lape et al., 2008; Mukhtasimova et al., 2005; Shi et al., 2023). The longitudinally-decreasing, coarse gradient in Φ suggests that the channel-opening gating transition is a conformational cascade (‘wave’) that propagates from the agonist to the gate, block-wise (Grosman et al., 2000), perhaps as an extended LFER. To emphasize that in AChRs binding ispart of gating, we show that a CRC and synaptic decay time constant can be calculated from the agonist association rate constant, k_on_ to C (catch) if η and L_0_ are known a priori. (**A**) CRC. For many agonists, k_off,C_ ~15,000 s^–1^ (Jadey and Auerbach, 2012). Calculate (i) K_dC_~1.5 × 10^4^ s^–1^/k_on,C_, (ii) K_dO_ (Equation 2), (iii) L_2_ (Equation 1) and (iv) P_O_^max^ and EC_50_ (Equation 4a, Equation 4b, Equation 4c, Equation 4d). For example, *η*=0.5 and L_0_=7.4 × 10^–7^ (at V_m_=-100 mV). ACh: measure k_on,C_=10^8^ M^–1^s^–1^, calculate K_dC_ = 150 μM, K_dO_ = 22 nM, L_2_=33, PO_m_^ax^ = 0.97 and EC_50_=31 μM. Choline: measure k_onC_ = 5 × 10^6^ M^–1^s^–1^, calculate K_dC_ = 3 mM, K_dO_ = 9 μM, L_2_=0.08, PO_m_^ax^ = 0.08 and EC_50_=6.8 mM. The procedure can be reversed (k_onC_ can be estimated from a CRC). (**B**) Synaptic decay time constant (τ). In adult-type AChRs the diliganded channel-closing rate constant (**b_2_**) for many agonists is ~2500 s^–1^ (–100 mV and 23 °C) (Grosman et al., 2000). *τ*~0.4 (1+f_2_/2*k_off_), where f_2_ is the diliganded opening rate constant, f_2_=L_2_*2500 s^–1^. Using the above k_on,C_ for ACh yields *τ*=1.5ms. That the agonist’s association rate constant can approximate P_O_^max^, EC_50_ and τ demonstrates the entanglement between binding and gating. Unlike diffusion, natural selection can adjust the catch ‘induced fit’ (k_on,C_) to fine tune physiological responses. ^1^Experimental evidence for catch in AChRs: k_on_ to C is (i) slower than the limit set by diffusion, (ii) correlated with agonist potency rather than diffusion constant, (iii) slower than k_on_ to O that is approximately diffusional (Nayak and Auerbach, 2017)^3^ and (iv) for choline highly temperature dependent (Gupta et al., 2017). Evidence that the hold stage of the induced fit occurs at the start of the global isomerization is that (i) *Φ*~0.95 for agonists and binding site residues (Purohit et al., 2013) (see below), and (ii) agonists increase the channel-opening rate constant (the affinity increase occurs before the transition state; Figure 1A). ^2^ Φ is log f_2_/log L_2_ for a series of perturbations and reports the free energy change of the perturbed location at the gating transition state (relative to A_2_O). In AChRs, a longitudinal, blocky, decreasing gradient in Φ (neurotransmitter site to gate) suggests the allosteric transition is a cascade of discrete domain rearrangements that connects A_2_C and A_2_O (Auerbach, 2005). Although Φ values sequence (1–0, early to late) and locate gating rearrangements, they do not provide temporal information or quantify energy coupling between domains. ^3^ The barrier that prevents agonists from forming AC_LA_ by diffusioserves a purpose. In AChRs, k_on,C_ correlates with agonist potency (Jadey and Auerbach, 2012; Jadey et al., 2011; Nayak and Auerbach, 2017), so the weak-agonist choline (present at the synapse at a high concentration) that would otherwise interfere with signaling is excluded from the pocket, preventing competitive antagonism. Extracellular cations compete with agonists to slow k_on,C_ (Cs^+^>K^+^>Na^+^>Li^+^) (Akk and Auerbach, 1996), and the mutation εE184Q eliminates this competition (Akk et al., 1999). We hypothesize that the ions and agonist compete at the encounter complex site (for instance, K^+^+C⇄^K^C) rather that the aromatic pocket. Agonist occupancy of the ultra-low-affinity ^A^C binding site triggers the catch-and hold rearrangement.

In bind, the agonist diffuses to the target and forms an encounter complex (Held et al., 2011; Homans, 2007; Schiebel et al., 2018), after which a local rearrangement (an induced fit) called ‘catch’ establishes the LA complex (A+C⇄^A^C⇄AC_LA_). Gate starts with the second stage of the induced fit called ‘hold’ that increases agonist affinity and, after several additional conformational changes in other protein domains, terminates with rearrangements in the pore that allow water and ions to pass (AC_LA_⇄AC_HA_⇄…⇄AO_HA_).

According to Equation 2, efficiency depends only on the LA/HA binding energy ratio. Regarding ΔG_LA_ (proportional to logK_dC_), the agonists we examined (Figure 5) all have approximately the same diffusion constant so we surmise that differences are caused by differences in catch. Regarding ΔG_HA_ (proportional to logK_dO_), the effect of perturbations away from the neurotransmitter site are agonist independent so we surmise that differences are caused by differences in hold. Therefore, with regard to η and the CRC (red pathway, Figure 1B), we attribute differences between agonists exclusively to differences in the free energy changes associated with induced fit that is the pair of catch-hold rearrangements, ^A^C⇄AC_LA_⇄AC_HA_ (Figure 2B).

The energy change in catch is ΔG_LA_ and in hold is ΔG_HA_-ΔG_LA_. Their sum, ΔG_HA_, is the total free energy change delivered by the ligand. From Equation 2, η is the fraction of this total associated with hold, and 1-η is the fraction associated with catch. Multiplying η by 100% gives the percent of agonist binding energy used for the hold rearrangement of the binding site that jumpstarts the full conformational cascade that connects the neurotransmitter sites with the gate.

In a pure binding reaction, for example A+C⇄^A^C, K_d_ is the concentration where the energy gained from formation of the complex is equal to the entropy lost from removing a ligand from solution that is indexed to a reference concentration (Phillips, 2020). However, if K_dC_ and K_dO_ are dominated by free energy changes associated with the induced fit, the entropy components become negligible (Figure 7—figure supplement 1). As far as comparative agonist action in AChRs is concerned, it appears that only the energy changes in catch and hold are germane.

**Figure 3.**
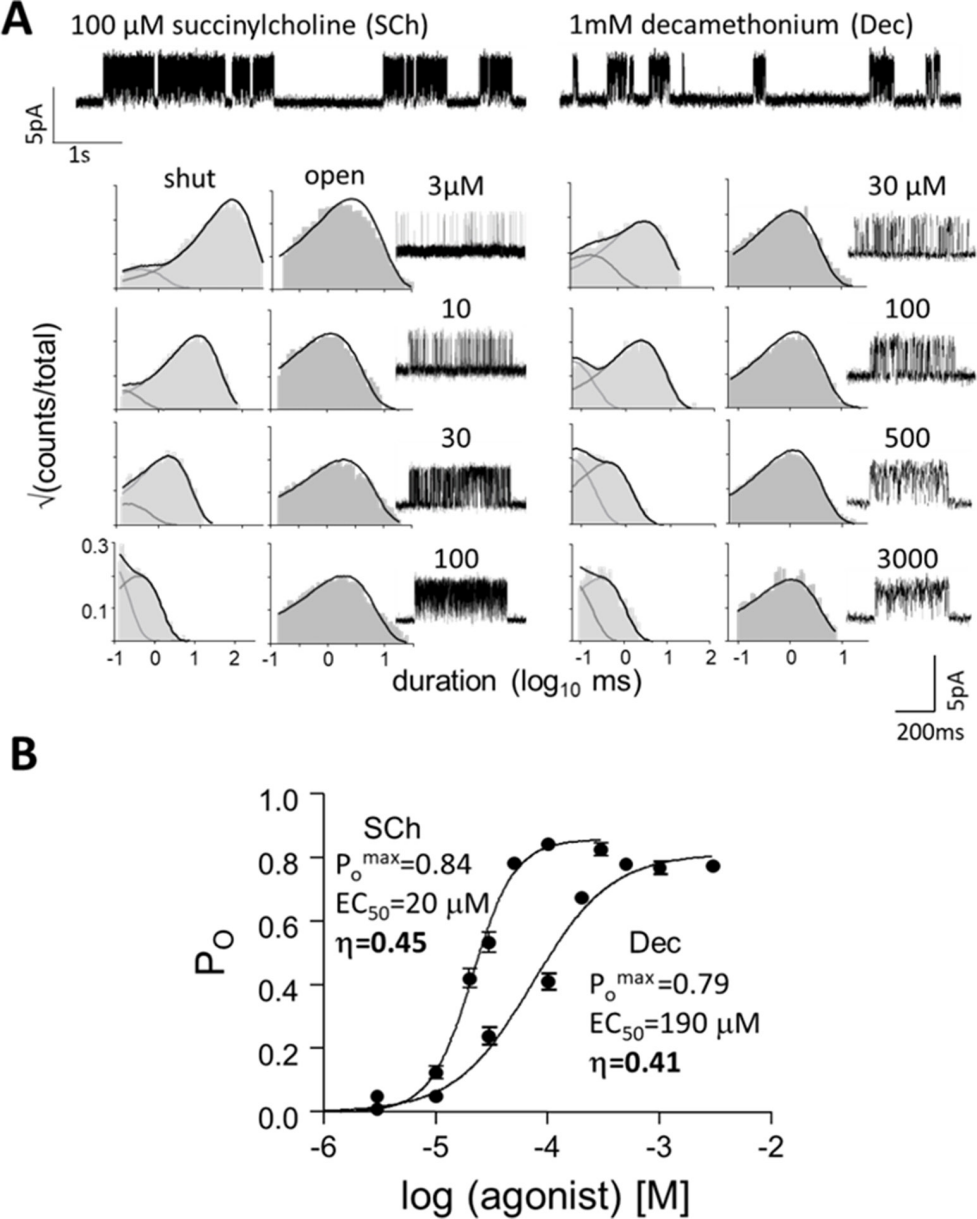
Measuring η. (**A**) Top, single-channel current traces, low time-resolution (V_m_ = +70 mV; O is up). Clusters of openings are bind-and-gate (Figure 1B); silent periods between clusters are desensitized. Bottom, example clusters and intra-cluster interval duration histograms. P_O_ was calculated at each [agonist] from shut- and open-interval time constants. (**B**) CRCs. P_O_ values were fitted to estimate P_O_^max^ and EC_50_ from which K_dC_ and K_dO_ were calculated (Equation 4a, Equation 4b, Equation 4c, Equation 4d, Materials and methods) (Table 1). The logs of these constants are proportional to ΔG_LA_ and ΔG_HA_, the ratio of which gives η (Equation 2). The profile for SCh is relatively left-shifted because this agonist is ~10% more efficient than Dec. Symbols are mean ± sem (Table 1; see Figure 3—figure supplement 1 for other agonists). The background mutation εS450W compensates for the effects of depolarization on the gating rate constants (see Materials and methods).

EC_50_ and P_o_^max^ were measured from each CRC (intra-cluster interval duration histograms in Figure 3 and Figure 3—figure supplement 1). K_dC_ and K_dO_ were calculated (Equation 4a, Equation 4b, Equation 4c, Equation 4d) after correction for background mutations that only changed L_0_. c, coupling constant (K_dC_/K_dO_); η, efficiency (Equation 2); n, number of CRCs. Membrane potential,+70 mV (to minimize channel block by the agonist). Agonist structures are in Figure 5; superscripts indicate mutation backgrounds: ^a^εS450W, ^b^εL269F, ^c^εE181W that increase L_o_ (to increase responses to weak agonists).

**Figure 3—figure supplement 1.**
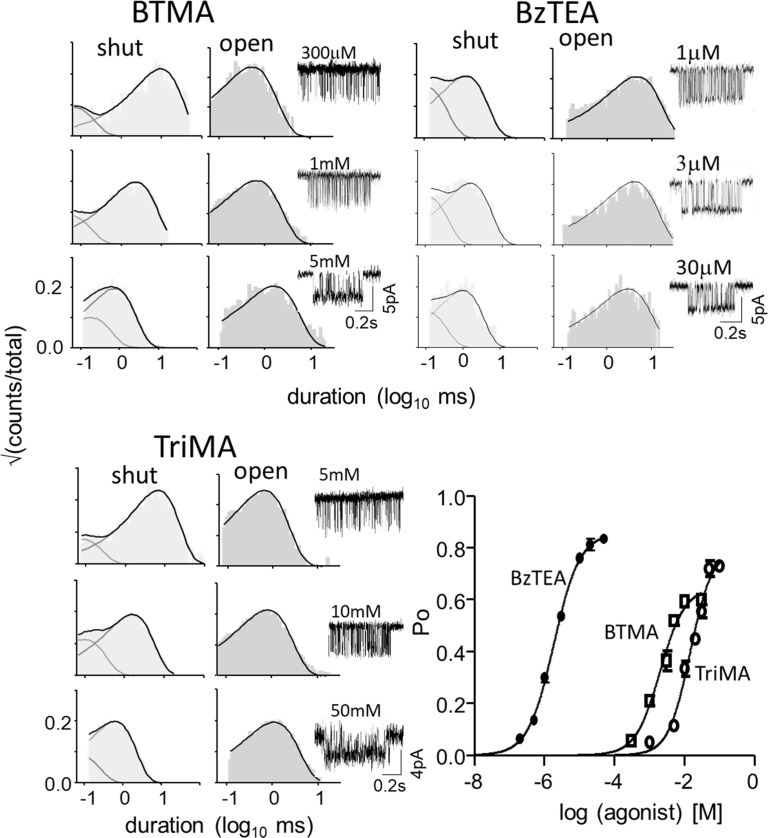
CRCs. Left, example histograms and clusters (see also text Figures 3 and 5). V_m_ = +70 mV; O is up. Efficiencies and background mutations are in Table 1. Symbols are mean + sem.

### Agonists

We measured η for 5 agonists using adult-type AChRs with wt neurotransmitter binding sites (Table 1). For each, single-channel currents were recorded at different agonist concentrations and P_O_ values calculated from shut and open interval durations were compiled into a CRC. L_0_ was known a priori (Nayak et al., 2012) so K_dC_ and K_dO_ could be calculated from EC_50_ and P_O_^max^ by using Equation 4a, Equation 4b, Equation 4c, Equation 4d (Materials and methods). As shown elsewhere, CRCs compiled from whole-cell currents serve equally well for η estimation (Indurthi and Auerbach, 2021).

Figure 3 shows CRCs for succinylcholine (SCh) and decamethonium (Dec). Although P_O_^max^ is similar for both agonists, EC_50_ (potency) of SCh is substantially lower than that of Dec. For each agonist, ΔG_LA_ and ΔG_HA_ were calculated to yield an η value (Equation 2) with the result were η_SCh_=0.45 and η_Dec_=0.41 (Table 1). SCh is 10% more efficient than Dec, which is the root cause of the abovementioned mismatch between P_O_^max^ and potency. Figure 3 shows that given L_0_, agonist efficiency can be calculated from a single CRC. Because η is the ratio of two logarithms, error arising from errors in P_O_^max^ and EC_50_ are small (Indurthi and Auerbach, 2021). CRCs for the other agonists are in Figure 3—figure supplement 1.

**Figure 4.**
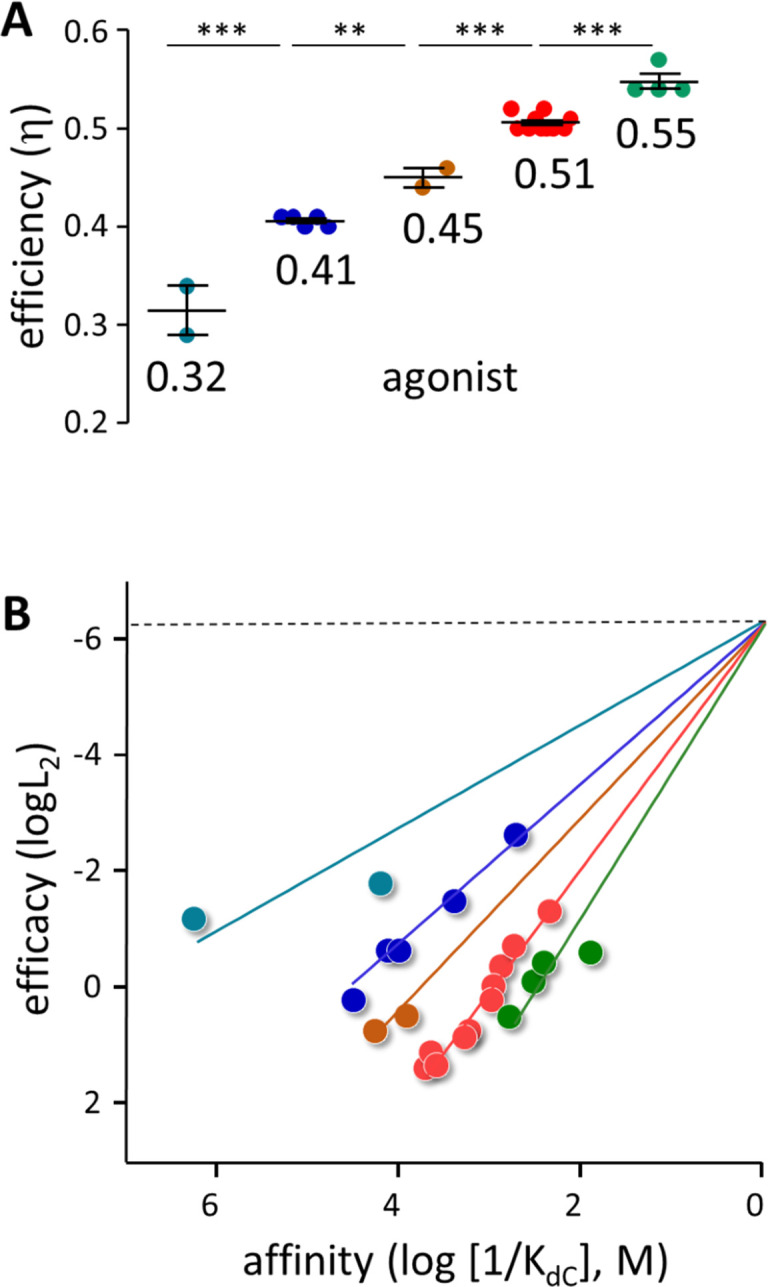
Agonist h values. (**A**) Each symbol represents the average η value of one agonist, calculated from CRCs (Table 1, Table 1—source data 1). The x-means algorithm was used to separate the agonists into 5 η classes (mean, vertical bars are ± sd). (**B**) Efficiency plots. Each color group was fitted by a straight line (Equation 3) with L_0_=5.2 × 10^–7^ (sd of each point smaller than the symbol). η values calculated from the slopes are the same as in panel A. x- and y-axes are proportional to the agonist’s free energy changes in catch (ΔG_LA_) and hold (ΔG_HA_-ΔG_LA_) induced fits (Figure 2B).

η values were calculated from previous measurements of K_dC_ for 18 other agonists (Figure 4A). An x-means cluster analysis of all 23 agonist η values indicates that there are 5 groups (mean ± sd): 0.32±0.035, 0.41±0.005, 0.45±0.014, 0.51±.008 and 0.55±0.015.

**Figure 5.**
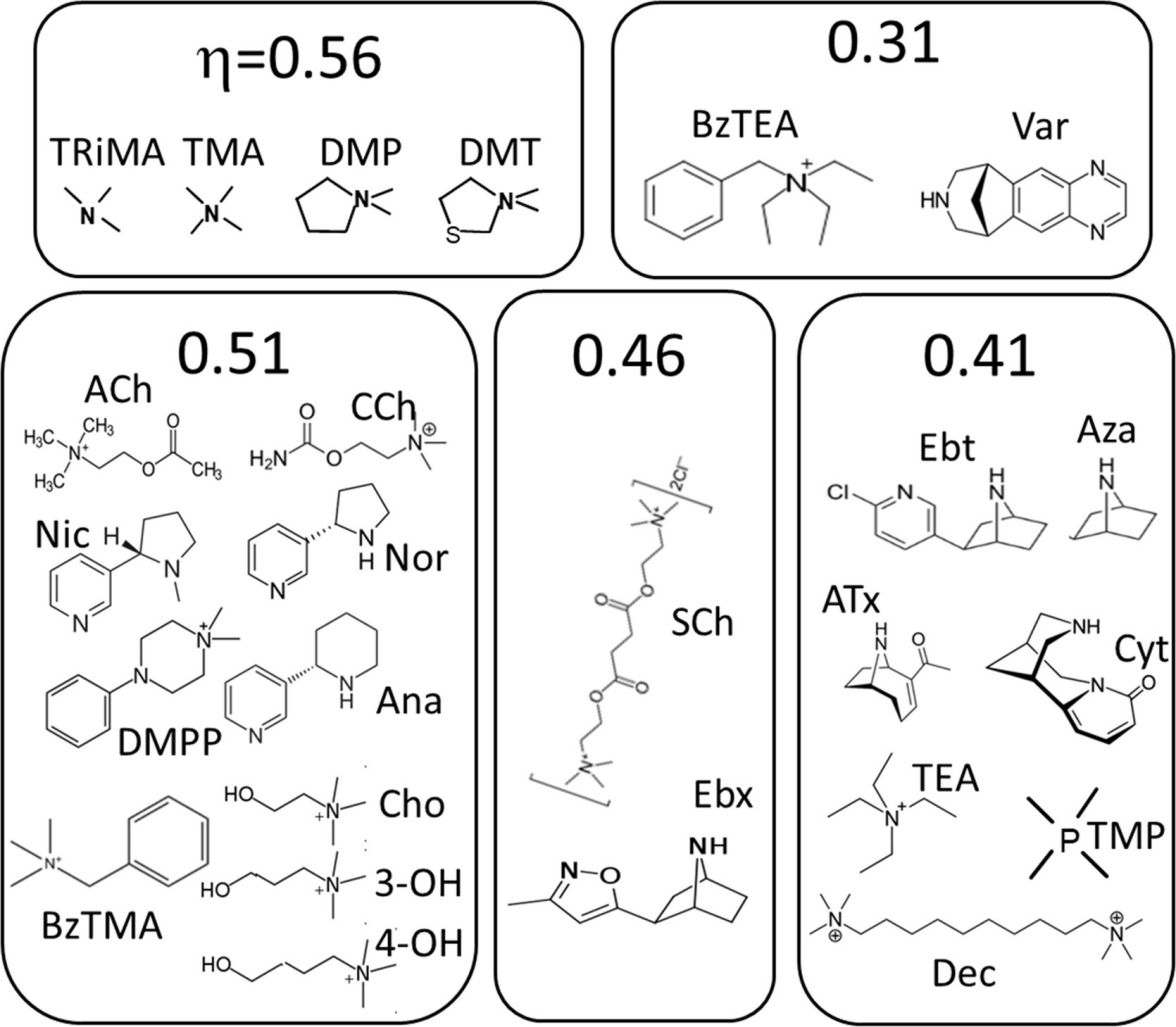
Agonists grouped by η class (Figure 4). See Materials and methods for abbreviations.

Figure 5 shows the agonists grouped by η class. The neurotransmitter ACh, its breakdown product choline (Cho), and the partial agonists carbamylcholine (CCh) and nicotine all belong to the *η*~0.51 class. The second-most common class, *η*~0.41, includes ligands that have a bridge nitrogen (for instance, Ebt) as well as others that do not (for instance, Dec and TMP). Small ligands (for instance, TriMA, MW 60) appear to be the most efficient and large rigid ligands (for instance, varenicline, MW 211) appear to be the least efficient.

It is valuable to combine Equations 1 and 2 (Nayak et al., 2019),

(3) $${logL}_{2}={logL}_{0}+mlog(\frac{1}{K_{dC}})\;\;\;\;\;\;\;\;\eta =\frac{m}{\left(m+2\right)}.$$

Equation 3 describes an ‘efficiency’ plot, log affinity (1/K_dC_) versus log relative efficacy (L_2_). An average η for a group of agonists is estimated from the slope of the straight line fit (m). Equation 4a, Equation 4b, Equation 4c, Equation 4d converts readily measured CRC parameters (P_O_^max^ and EC_50_) into equilibrium constants (K_dC_ and L_2_), and Equation 3 converts these into fundamental constants that pertain to the agonist (η) and the receptor (L_0_). The value of the efficiency plot is that it increases the accuracy of the η estimates because if L_0_ is known a priori*,* the y-intercept can be added as a fixed point to all lines. For receptors in which L_0_ has not been measured, the efficiency plot offers a convenient way to do so, as was done previously for glutamate, GABA, glycine, and muscarinic recepotors (Nayak et al., 2019).

η values estimated from the slopes for the 23 agonists (Figure 4B) are the same as from the x-mean cluster analysis (Figure 4A) (mean ± sd): 0.31±0.018, 0.41±0.002, 0.46±0.004, 0.51±0.002 and 0.56±0.008. Affinity and efficacy are correlated significantly within the 3 classes having >2 members (19 agonists; Pearson’s correlation test p-values for *η*=0.41 and 0.51,<0.0001, and for *η*=0.56, 0.019). Also, the slopes for these 3 classes are significantly different (ANCOVA, *F*=129, p<0.001). The association of the other agonists with a discrete η class is less certain but supported by mutation studies (see below).

Figure 4B shows that in AChRs, agonists having the same affinity can have different efficacies, and *vice versa*. Absent classification of an agonist according to η (imagine all symbols the same color) there is no global correlation between these two agonist properties. However, a linear correlation between log affinity and log efficacy is clear within each class. In AChRs, the presence of multiple η classes precludes a global correlation between efficacy and affinity.

### Mutations

**Figure 6.**
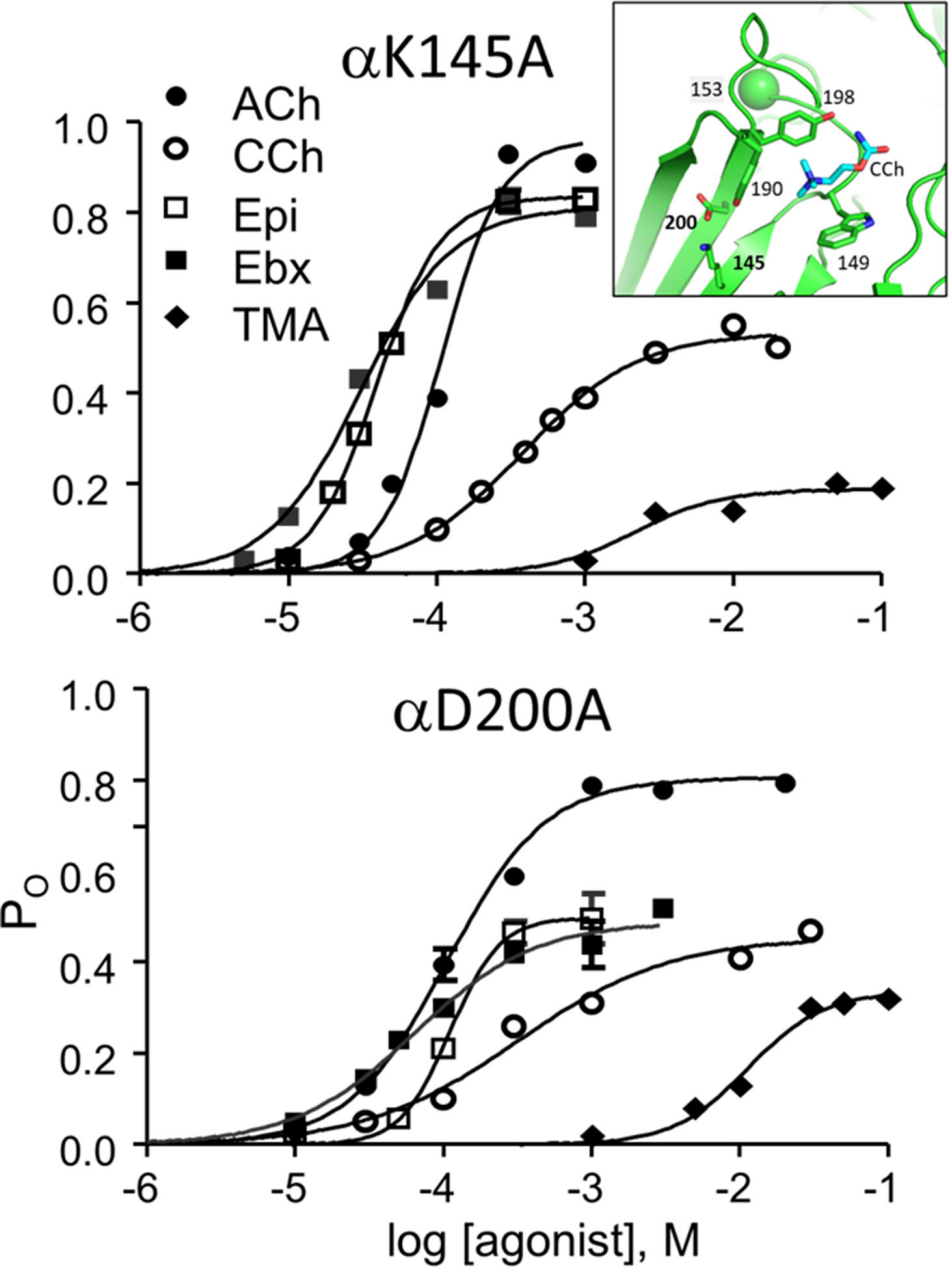
CRCs for αK145A and αD200A. Agonist efficiencies calculated from the fitted CRC parameters are in Table 2. Intra-cluster interval histograms are in Figure 6—figure supplement 1 and Figure 6—figure supplement 2. Inset, α−δ subunit interface of an AChR neurotransmitter binding site occupied by CCh (cyan) (7QL6.pdb; Zarkadas et al., 2022).

η values were measured previously in adult-type AChRs having one of 42 binding site mutations (Table 2—source data 1). To these we add 11 more (Table 2), for 5 agonists and 3 binding-site mutations (αD200A, αK145A, αG153S; Figure 6, Figure 6—figure supplement 1, Figure 6—figure supplement 2).

**Figure 6—figure supplement 1.**
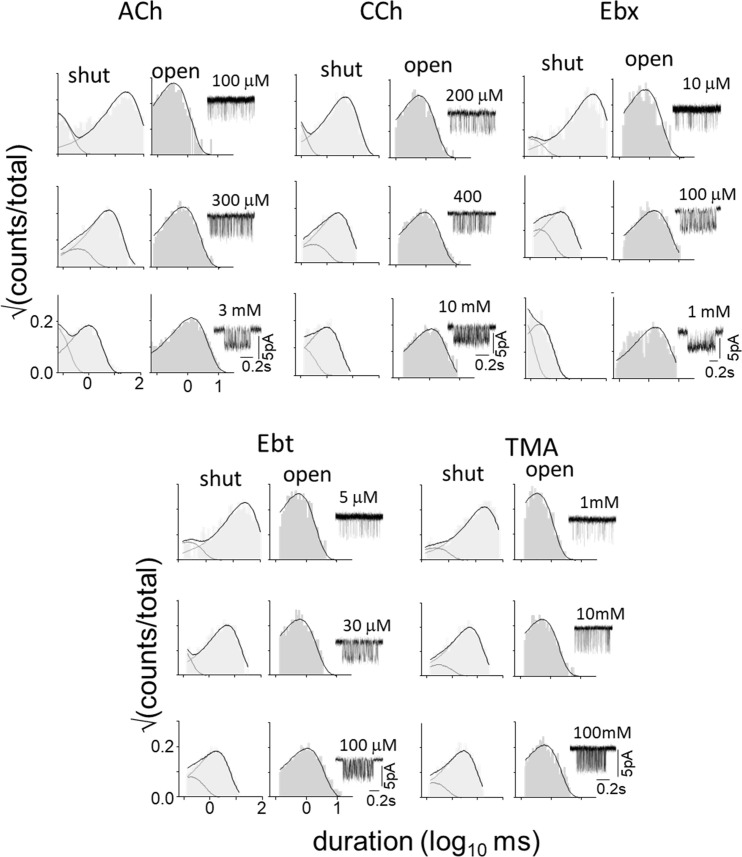
αK145A. Example histograms and clusters (agonist structures in Figure 5). V_m_ = +70, O is up. CRCs are in Figure 6; efficiencies and backgrounds are in Table 2.

**Figure 6—figure supplement 2.**
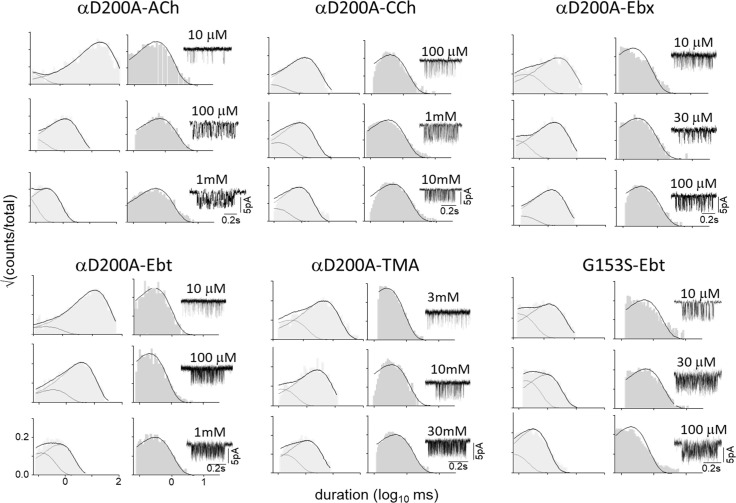
αD200A and αG153S. Example histograms and clusters (agonist structures in Figure 4). V_m_ = +70, O is up. CRCs in Figure 6. (Efficiencies and backgrounds in Table 2).

**Table 2.** Mutation efficiencies. Table 2—source data 1.Mutation efficiency.

mutation	agonist	EC_50_ (μM)	P_O_^max^	K_dC_ (μM)	K_dO_ (nM)	c	η^mut^	n	η^wt^
D200A	ACh^a^	110 (10)	0.80 (0.02)	52 (3)	150 (5)	338 (12)	0.37 (0.00)	3	0.50
	CCh^a^	320 (70)	0.45 (0.02)	138 (18)	900 (94)	153 (3)	0.36 (0.00)	4	0.52
	TMA^a^	13350 (2970)	0.37 (0.03)	5683 (740)	4382 (890)	130 (5)	0.48 (0.02)	5	0.54
	Ebt^a+b^	110 (20)	0.50 (0.03)	47 (5)	680 (47)	69 (2)	0.30 (0.00)	3	0.41
	Ebx^a+b^	060 (20)	0.49 (0.03)	26 (5)	390 (58)	67 (3)	0.29 (0.01)	3	0.46
K145A	ACh^a^	110 (10)	0.95 (0.06)	83 (5)	50 (3)	1672 (82)	0.44 (0.00)	3	-
	CCh^a^	380 (30)	0.53 (0.01)	165 (8)	400 (15)	411 (5)	0.41 (0.00)	4	-
	TMA^a^	2200 (590)	0.19 (0.01)	922 (125)	5010 (712)	184 (3)	0.43 (0.01)	3	-
	Ebt^a^	40 (4)	0.81 (0.04)	20 (2)	20 (2)	792 (52)	0.38 (0.00)	3	-
	Ebx^a^	40 (3)	0.83 (0.01)	20 (1)	20 (1)	847 (17)	0.38 (0.00)	3	-
G153S	Ebt^a^	2 (0.5)	0.65 (0.01)	2 (0.3)	7 (1)	313 (4)	0.31 (0.01)	4	-

Measured EC_50_ & P_o_^max^ and calculated K_d_, c and h, mean (sem). For mutation location see Figure 6, inset. K_dC_ and K_dO_ were calculated from CRC parameters (Figure 6) by using Eq. 4. c, coupling constant (K_dC_/K_dO_); n, number of CRCs. L_0_ was corrected for background mutations (Methods): ^a^εS450W, ^b^εL269F, ^c^εE181W. See Figure 6—figure supplement 1 and Figure 6—figure supplement 2 for intra-cluster interval duration histograms.

αD200 and αK145, along with αY190, have been suggested to work together to initiate the channel-opening conformational change (Mukhtasimova et al., 2005). The mutation αY190A reduces η_ACh_ from 0.50 to 0.35, but αY190F is without effect (Bruhova and Auerbach, 2017). However, αY190F does cause substantial losses in LA and HA binding energies for ACh, to an extent that depends on the αK145 side chain (Bruhova and Auerbach, 2017). These results support the suggestion that these side chains work together, but exactly how and to what effect remains unclear. The agonists we tested with αD200A or αK145A were from 4 different η classes (wt class value): TMA (0.54), CCh (0.51), ACh (0.50), Ebx (0.46), and Ebt (0.41).

For mutation location see Figure 6, inset. K_dC_ and K_dO_ were calculated from CRC parameters (Figure 6) by using Equation 4c, coupling constant (K_dC_/K_dO_); n, number of CRCs. L_0_ was corrected for background mutations (Materials and methods): ^a^εS450W, ^b^εL269F, ^c^εE181W. See Figure 6—figure supplement 1 and Figure 6—figure supplement 2 for intra-cluster interval duration histograms.

The results are in Table 2. The substitution αD200A reduces the efficiency of 4 of the 5 agonists to 0.33±0.04 (mean ± sd), or to about the same level as with αY190A with ACh. The exceptional ligand was TMA for which η remained relatively high at 0.48. In contrast, in αK145A η for all 5 agonists was 0.41±0.03. Interestingly, this mutation reduces η for ACh, CCh and TMA but not significantly for Ebx and Ebt. Note that the mutation αW149A increases η for ACh to 0.60 (Purohit et al., 2014).

Table 2—source data 1 shows K_dC_ and K_dO_ values measured previously for 4 different agonists in AChRs having a substitution at αG153 (Jadey et al., 2013), including a Ser that causes a congenital myasthenic syndrome (Engel et al., 1982). After converting the published equilibrium constants to ΔG_LA_ and ΔG_HA_, the results indicate that on average the mutations reduce η for Cho, DMP, TMA and nicotine to 0.41±0.03 (mean ± sd), or by ~25%. To these we add the new result that αG153S reduces η of Ebt from 0.42 to 0.31, or also by ~25%.

**Figure 7.**
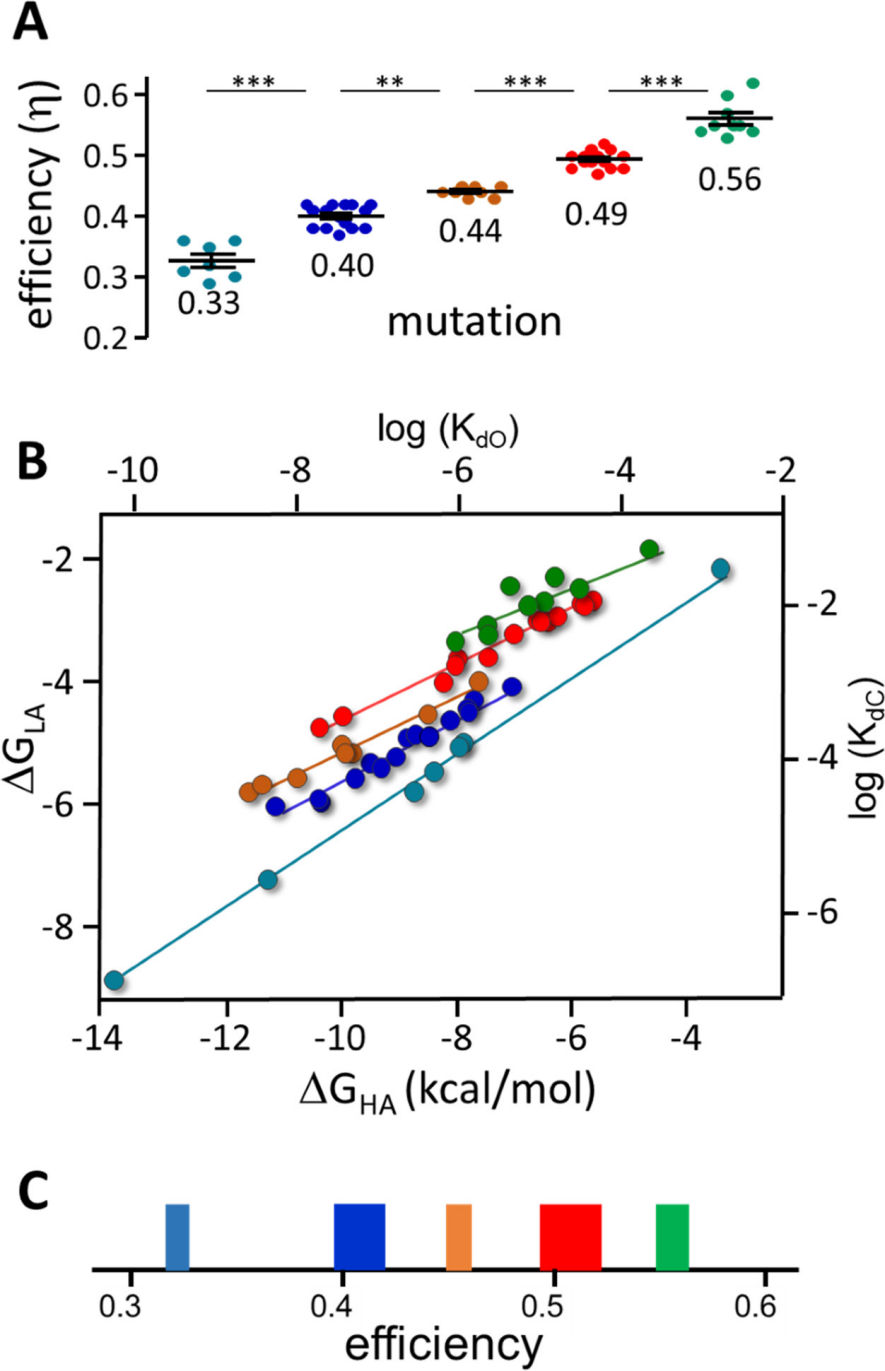
Mutation η values. Each symbol is η value calculated for an individual mutation (various agonists; Table 2 and Table 2—source data 1). (**A**) There are 5 efficiency classes (mean ± sd). As with agonists (Figure 4), the ~0.5 and~0.4 classes predominate. (**B**) Weak (LA) versus strong (HA) binding energies for the mutants (sd of each point is smaller than the symbol). The 5 slopes reflect different correlations between catch and hold free energy changes (Figure 2B). The efficiency distribution for the mutations (*η*=1-slope; see text for mean ± sd) is the same as for agonists. C. Spectrum of efficiencies, adult AChR neurotransmitter binding sites. The width of each line is proportional to prevalence (agonists and mutations).

Figure 7A shows x-means cluster analysis of efficiencies for 53 AChR binding site mutations. Mutant η values (mean ± sd) segregate into the same 5 η classes that were apparent with agonists: 0.33±0.03, 0.40±0.02, 0.44±0.01, 0.49±0.01 and 0.56±0.03. The η classes that were under-represented and poorly defined with agonists (Figure 4B) are more common and clearer with mutations.

**Figure 7—figure supplement 1.**
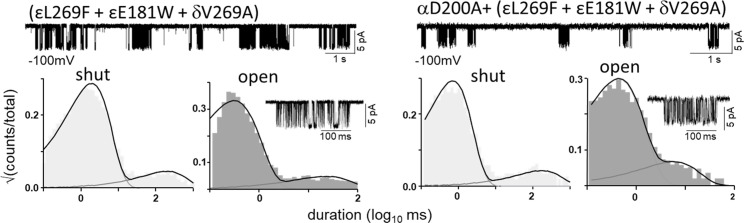
Estimating L_0_ (αD200). All currents were recorded in the complete absence of agonists. Top traces, clusters of unliganded single-channel openings are mainly C⇄O (V_m_=-100 mV; O is down); silent periods between clusters are desensitized. Bottom, interval duration histograms and an example cluster. Left, the background mutations together increase L_0_ from 7.4x10^–7^ to 0.17 (by a factor of 2.46x10^5^). Right, the background mutations plus αD200A increase L_0_ from 7.4x10^–7^ to 0.64 (by a factor of 3.76x10^5^). Hence, the A substitution at αD200 increases L_0_ by 3.76-fold. L_0_ is voltage dependent and is 5.2x10^–7^ at –70 mV (Nayak et al., 2012). We calculate that at this membrane potential (Figure 6) L_0_ with αD200A is 3.76-fold grater, or 1.9x10^–6^. L_0_ was measured individually using the above method for all 53 mutations (Table 2 and Table 2—source data 1).

**Figure 7—figure supplement 2.**
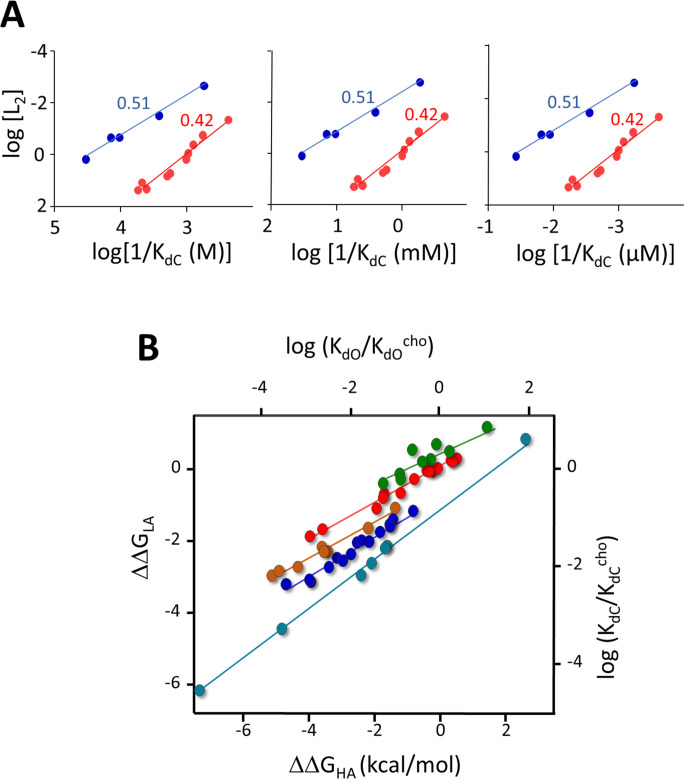
Evidence that in AChRs K_dC_ reflects mainly the free energy change of the catch rearrangement (see Figure 2—figure supplement 1). In A+C⇄^A^C ⇄AC (Figure 2A), the first step includes a term for the entropy decrease from the loss of a free ligand, indexed to a reference concentration (Phillips, 2020). This term does not cancel in Equation 2, but in AChRs the second step dominates so η is the same with or without this term. (**A**) Agonist η-plots (0.51 and 0.42 class ligands; Figures 4B and 5) using different reference concentrations. The slopes of the straight-line fits and η (Equation 3) are independent of the reference concentration. (**B**) Mutation plots. The entropy term was removed by first normalizing K_dC_ and K_dO_ by those of a standard agonist (here, choline; 2 other agonists give the same result). The slopes of plots, ΔΔG_LA_/ΔΔG_HA_, are approximately same as without normalization (Figure 7B).

With mutations, an η-plot is not useful because substitutions can change L_0_ (see Figure 7—figure supplement 1) and, hence, the y-intercept of each line. Instead, a plot of ΔG_LA_ versus ΔG_HA_ shows the binding energy correlations directly. This plot for mutations (Figure 7B) shows 5 slopes (Pearson’s correlation test p-value <0.0006 for all classes). The distribution of η values (1-slope) is (mean ± sd): 0.32±0.007, 0.40±0.004, 0.44±0.003, 0.49±0.002 and 0.56±0.007, with all slopes being significantly different (ANCOVA, F-value, 63.35; p-value,<0.0001).

Combining the results for agonists and mutations, the overall distribution of η (relative prevalence) is: 0.56 (17%), 0.51 (31%), 0.45 (13%), 0.41 (26%), and 0.31 (12%). As was the case with agonists and mutations separately, the 0.51 (example, ACh) and 0.41 (example, Ebt) classes predominate. Figure 7C shows the distribution of agonist plus mutation η values as a spectrum in which line thickness represents relative prevalence.

## Discussion

### Efficiency

Efficiency is the missing link that connects binding to gating (Equation 2). Insofar as K_dC_ and K_dO_ apply generally (Figure 1), η is a universal agonist attribute that depends only on these two constants. In AChRs, K_dC_ and K_dO_ are set mainly by energy changes in a pair of local rearrangements of the binding site (the catch-and-hold induced fit), with η being the fraction of the total used to initiate the allosteric transition of the receptor. As such, η calibrates the fundamental connection that defines receptor action.

Converting ligand binding energy into energy for an otherwise unfavorable protein conformational change is an induced fit. We assume that without an agonist present, both catch and hold rearrangements (of an aromatic pocket) are energetically unfavorable and generate the high barrier to unliganded opening (Figure 1). In enzymes, a fraction of substrate binding energy is used to promote a local protein rearrangement that stabilizes the reaction transition state (Richard, 2022). In AChRs, neurotransmitter binding energy is divided equally between two stages of an intrinsically unfavorable rearrangement that forms the LA and HA complexes and starts the gating isomerization. In brief, η quantifies the split in ligand binding energy between the two steps in the catch-and-hold induced fit (Figure 2B).

### η classes

Agonists having radically different resting affinities and efficacies can have the same η. For example, ACh (K_dC_ = 175 μM, PO_m_^ax^ = 0.96) and choline (K_dC_ = 4 mM, PO_m_^ax^ = 0.05) both divide their binding energy equally between catch and hold. In AChRs, agonists segregate into discrete ηclasses within which all members use approximately the same fraction of their post-diffusion binding energy for catch. So far we have identified 5 η classes with catch percentages ranging from 47% to 71% (Table 1). Below, we discuss the possibility that larger ligands apply a larger percentage of their binding energy to catch.

η classes are notable because its two component energy changes (Equation 3) are realized in bind and in gate, usually considered to be qualitatively different processes (Figure 1B). Nevertheless, the existence of an η class indicates that in AChRs these two binding energy (structure) changes are correlated and joined in a linear free energy relationship (LFER) (Figure 2B). Any change in bind (catch) free energy compels one in gate (hold), reciprocally. Rather than being independent processes, bind and gate are completely entangled even if they are depicted as different dimensions of a reaction scheme (Figure 1B).

Although an empirical η value can be calculated for every agonist of every receptor, the generality of η classes is less certain. The components, K_dC_ and K_dO_ for many agonists, have not been measured extensively in other receptors, but some results suggest that a catch-and-hold induced fit, with stages linked in a LFER, is not uncommon.

Regarding hold, it is likely that an increase in affinity caused by a local binding site rearrangement inside the gating isomerization is typical (AC_LA_⇄AC_HA_). In enzymes and receptors, agonists induce a ‘clamshell’ closure of the ligand site that is associated with increased binding strength (Armstrong and Gouaux, 2000; Masiulis et al., 2019; Pless and Lynch, 2009; Traynelis et al., 2010). Also, in many receptors agonists increase the opening rate constant as well as P_O_ (Clements et al., 1998; Clements and Westbrook, 1991; Maconochie et al., 1994) indicating that the stabilization by the lgiadn of an otherwise unfavorable rearrangement (the affinity increase) occurs early in the isomerization (Figure 1A). Finally, in other receptors an AC_HA_ intermediate state has been detected directly in single-channel currents (Lape et al., 2008; Shi et al., 2023) or identified in structures (Sauguet et al., 2014).

Regarding catch, this first step in the induced fit serves a purpose (Figure 2—figure supplement 1) and may also pertain to other receptors. As in AChRs, in several receptors k_on_ to C is slower than diffusion and correlated with agonist potency (Dravid et al., 2008; Grewer, 1999; Lewis et al., 2003; Mortensen et al., 2010). Further, a catch-and-hold LFER is implied in other receptors (including a GPCR; Sykes et al., 2009) because efficiency plots are linear, with outliers that possibly indicate different η classes (Nayak et al., 2019). In biological reactions, LFERs are empirical descriptors and more experiments are needed to determine the generality of η classes. In addition to measurements of K_dC_ and K_dO_ in other receptors, and identifying the structures of intermediate liganded states ^A^C and AC_LA_ would corroborate catch.

In AChRs, multiple η classes together preclude the appearance of a global affinity-efficacy correlation. If η classes turn out to be common, then the consensus view that there is no correlation between these agonist properties needs to be reexamined (Colquhoun, 1998; Kenakin and Onaran, 2002). The clear correlation between log affinity and log efficacy within each class vanishes when agonists from different classes are lumped together (Figure 4B).

We detected 5 η classes in AChRs but additional experiments could reveal more. In particular, agonist classes with the highest and lowest η values have the greatest scatter and could be amalgams. Nonetheless, the ability to corral 76 different agonist-receptor combinations into just 5 classes represents a significant reduction in complexity. If nothing else, η is a useful way to classify agonists and, possibly, binding sites. Also, η could prove to be useful CRC analysis because it allows EC_50_ to be computed from P_O_^max^ and *vice versa* by using (Equation 3, Equation 4a, Equation 4b, Equation 4c, Equation 4d; Indurthi and Auerbach, 2021).

Mutations of binding site residues segregate into the same 5 efficiency classes as do agonists. This supports the idea that an agonist at a binding site is much like an ordinary side chain, with exceptions. Most importantly, agonists are not linked covalently and so are both hypermobile in the pocket and free to come and go to serve as a signal. Also, by definition a bound agonist is more stable in O versus C (Figure 1A) but this is the opposite of most AChR wt side chains (Purohit et al., 2013). η measurements reinforce the standard view that ligands only perturb the intrinsic activity of allosteric proteins.

### η and structure

In AChRs, the pair of structural changes at the binding sites associated with energy changes in hold (ΔG_HA_-ΔG_LA_) and in catch (ΔG_LA_) are not known with certainty. Although there are structures of apo-C (Zarkadas et al., 2022) and desensitized (perhaps the same as AO_HA_) (Auerbach, 2020; Rahman et al., 2020; Zarkadas et al., 2022), those of the three relevant liganded-closed intermediate states ^A^C, AC_LA_ and AC_HA_ are not available. Here, we discuss inferences about the structures of these three transient states that can be made from η.

If every agonist had a unique η then little could be said about the underlying conformational changes. However, the existence of an η class and, therefore, an LFER between catch-and-hold energy changes suggests that the corresponding structural changes, too, are correlated, although perhaps not linearly. That is, catch and hold are two related stages of a single induced fit rearrangement of the binding site.

Regarding hold, at HA sites loop C covers (‘caps’) the aromatic pocket (Nys et al., 2013) that appears to be contracted. Also, simulations suggest the possibilities that in the hold step the ligand flips its orientation (Tripathy et al., 2019), and that water is extruded from the binding interface (Young et al., 2007). The components of the hold free energy change arise from these and other restructuring events, but the breakdown is not known. Regarding catch, little is known about this rearrangement directly because the structures of neither end state have been solved. (Note that apo-C and ^A^C are different). However, because of the LFER, we hypothesize that all or some of the above putative rearrangements in hold (cap, contract, flip, de-wet) also happen in catch, but perhaps to lesser extents.

A surprising inference about the binding site structural change comes from measurements of agonist association rate constants (k_on_) to C versus O (Grosman and Auerbach, 2001; Nayak and Auerbach, 2017). Whereas k_on_ to C is slower than diffusion and correlated strongly with agonist potency, k_on_ to O is approximately at the diffusion limit and weakly agonist dependent. Hence, slow association to C cannot be attributed to the ligand itself but rather suggests that the hold stage of the induced fit removes a barrier(s) that otherwise prevents free entry of the ligand into the aromatic binding pocket (Figure 2—figure supplement 1). Neither the site of the encounter complex nor the nature of this barrier are known. Because loop C capping and pocket contraction would be expected to decrease rather than increase k_on_, we speculate that in AChRs other, still-unidentified structural changes in hold serve to remove the barrier. Removing loop C eliminates activation by agonists but not unliganded gating (Purohit and Auerbach, 2009). Apparently, clamshell closure is necessary for transducing ligand binding energy into the receptor isomerization, but not for the global isomerization itself. However, it is uncertain if other rearrangements in the binding site induced fit occur in unliganded gating after loop C removal (see below).

Energy change reflects a change in structure, so multiple η classes (logK_dC_/logK_dO_ ratios) imply there are multiple pairs of C versus O (post-catch and post-hold) binding site conformations. The existence of 5 discrete ΔG_LA_/ΔG_HA_ ratios indicates that there are at least this many AC_LA_/AC_HA_ binding site structural pairs. Possible combinations that give rise to 5 η classes are (number of AC_LA_/number of AO_HA_) 1/5, 2/3, 3/2, 4/2, and 5/1. For example, with the 1/5 combination a single AC_LA_ binding site structure selects its target AO_HA_ structure from among 5 alternatives.

The discrete distribution of η classes suggests that the structural changes in the two stages of the induced fit also are discrete rather than continuous. The AChR allosteric transition combines elements of induced fit (use of binding energy to drive a local rearrangement) and conformational selection (constitutive activity and the adoption of pre-existing target structures) (Changeux and Edelstein, 2005).

The binding energy increase (in hold) occurs at the onset of the channel-opening gating isomerization (Grosman et al., 2000). The existence of multiple, distinct C versus O structural pairs at each binding site indicates that the end states in the simple activation scheme (Figure 1B) do not represent single stable structures but rather ensembles made up of receptors having at least five distinct binding site structural pairs. This raises the possibility that there could be five isomerization pathways that might culminate in five different structural perturbations of the gate region. Although there is no evidence in AChRs of any agonist-dependent variations in output properties (conductance or ion selectivity), in other receptors functional output is agonist dependent (Kenakin and Christopoulos, 2013). Efficiency measurements of these receptors would test the possibility that η classes are associated with the phenomenon of biased agonism (Ehlert, 2018).

Finally, regarding the agonist (Figure 5), there is a trend for those with cationic centers that occupy smaller volumes *in vacuo* to have greater η (Indurthi and Auerbach, 2021). That is, agonists with larger volumes tend to use a greater fraction of their binding energy for catch. In AChRs and other receptors, the volume of the binding pocket appears to be smaller in O versus C (Tripathy et al., 2019), so it is also possible that unfavorable VdW interactions caused by pocket contraction guide the selection of (for example) the target O structure, to decrease η. The relationship between catch and hold energy change and the corresponding structural elements (agonist, protein, water) is complex and further investigations are needed.

### Extending η

The catch-and-hold LFER implied by an η class indicates that structure (energy) changes in these two binding site rearrangements are related. Microscopic reversibility demands that if catch promotes hold, then hold promotes catch. Below, we present evidence suggesting that this bidirectional linkage is the tip of an iceberg, and that the entire AChR activation cascade - from an agonist at the encounter complex to water at the gate (^A^C to AO) - is linked as an extended, multi-stage LFER (Figure 2—figure supplement 1).

There have been several proposals in AChRs regarding structural changes that link the neurotransmitter sites and the gate, but these are difficult to assess because they are without corroborating energy measurements. Likewise, η measurements reveal a binding-gating energy link at the agonist site, but lack corroborating structural information. However, separate activation domains in AChR activation have been identified. For instance, η calibrates the catch-hold connection at the agonist binding site, mutations decouple the ECD-TMD interface (Cymes and Grosman, 2021; Shi et al., 2023) and interactions between gate residues and pore water have been investigated (Beckstein and Sansom, 2006; Rasaiah et al., 2008; Yazdani et al., 2020). Analyses of the gating transition state suggest that there are 5 discrete domain rearrangements (Figure 2—figure supplement 1). In opening, hold is followed by movements of the ECD, TMD, gate region, and, finally, pore water and membrane lipids (Gupta et al., 2017).

Evidence for the extended LFER hypothesis is the experimental measurements of k_on_ to C versus O. As mentioned above, when an unliganded AChR opens constitutively, k_on_ increases to approach the diffusion limit, and also loses its agonist dependence. This indicates that with loop C intact, adopting the global O shape in the absence of agonists is sufficient to induce the local change in structure that removes the entry barrier, in hold. That a distant mutation (for example, of a gate residue) increases constitutive opening and also influences the ligand association rate constant is evidence that binding and gating are entangled.

This result, along with η and Φ measurements, leads us to propose that everything in the protein’s activation cascade, ^A^C to AO, is energy-linked in a reversible, extended LFER. In this sequence, each individual domain energy change (rearrangement) influences those of its neighbors, bidirectionally. Usually, the first link in activation (after the encounter complex) is catch-and-hold, and the last is gate-and-water. However, in a LFER the consequence of a perturbation propagates both forward and backward, so a gate mutation would also perturb the agonist site and one at the ECD-TMD would induce rearrangements both there and at the gate. An extended LFER offers a way to transfer energy (change structure) over distance by using only local domain rearrangements. In AChRs, the allosteric transition appears to be a energy-coupled chain of domain rearrangements that crosses seamlessly the traditional divide between binding and gating. η is at the core receptor operation insofar as it links the induced fit with the conformational gating cascade, Φ reveals the cascade’s components (and the sequence of domain rearrangements), and k_on_ to O reveals that a reverse cascade, gate→binding site, exists without agonists. Transit across the whole extended LFER is rapid (~5 μs; Lape et al., 2008), with each interemdiate states lasting on the order of 100 ns (Gupta et al., 2017).

There are many unanswered questions. We do not know the structural changes in the induced fit, or their energy consequences. Regarding agonists, we do not know the full spectrum of η classes, or their structural correlates. Regarding mutations, we do not know the reasons specific agonist/side chain combinations change η or whether mutations of additional residues (including at a distance from the agonist site) can also do so. Regarding binding sites, we know little about the location of the encounter complex, why agonists are more efficient at the fetal AChR neurotransmitter site (Nayak et al., 2019), or η values in other subtypes of nicotinic receptor or atl allosteric modulator binding sites. The extended LFER hypothesis needs to be tested, with both structure and energy changes identified and quantified. Regarding other receptors, there are few reports of the key experimental values (K_dO_, k_on_ to O, Φ) so it is unknown whether η classes, a staged induced fit, a conformational cascade, or an extended LFER apply generally. We anticipate that additional structure and energy measurements, in combination with computation, will reveal the molecular forces that underpin activation and lead to a deeper understanding of how ligands promote conformational change in allosteric proteins.

## Materials and methods

### Expression

Human embryonic kidney (HEK) 293 cells were maintained in Dulbecco’s minimal essential medium (DMEM) supplemented with 10% FBS and 1% penicillin–streptomycin (pH 7.4). HEK cells obtained from ATCC (CRL-1573, lot no. 57925149) are authenticated using STR profiling and tested free of mycoplasma contamination. Mutations were incorporated into AChR subunits using the Quickchange II site directed mutagenesis kit (Agilent Technologies, CA) according to manufacturer’s instructions. Sequence was verified by nucleotide sequencing (IDT DNA, I). AChRs were transiently expressed in HEK 293 cells by transfecting (CaPO_4_ precipitation) (Purohit et al., 2014) mouse α1 (GFP encoded between M3-M4),β1,δ,ε subunits (3–5 μg total/ 35 mm culture dish) in a ratio of 2:1:1:1 for ~16 hrs. Most electrophysiological experiments were done 24–48 hr post-transfection.

### Electrophysiology

Single-channel currents were recorded in cell-attached patches (23 °C). The bath solution was (in mM) 142 KCl, 5.4 NaCl, 1.8 CaCl_2_, 1.7 MgCl_2_, 10 HEPES/KOH (pH 7.4). High extracellular [K^+^] ensured that the membrane potential V_m_ was ~0 mV. Patch pipettes were fabricated from borosilicate glass, coated with sylgard (Dow Corning, Midland, MI) to a resistance of ~10 MΩ when filled with pipette solution (Dulbecco’s phosphate-buffered saline PBS) (in mM): 137 NaCl, 0.9 CaCl_2_, 2.7 KCl, 1.5 KH_2_PO_4_, 0.5 MgCl_2_, and 8.1 Na_2_HPO_4_ (pH 7.3/NaOH). Single channel currents were recorded using a PC505 amplifier (Warner instruments, Hamden, CT), low-pass filtered at 20 kHz and digitized at a sampling frequency of 50 kHz using a data acquisition board (SCB-68, National instruments, Austin, TX). For liganded activation experiments, agonists were added to the pipette solution at the desired concentrations. For unliganded activation experiments, we used pipettes and wires that were never exposed to agonists. To reduce the effect of channel block without affecting binding of agonist to the receptor, membrane potential (V_m_) was held at +70 mV when agonists were used (Jadey et al., 2011).

### Current analysis

Analyses of the single-channel currents were performed by using QUB software (Nicolai and Sachs, 2013). Single-channel currents occur in clusters when the opening rate constant is significantly large. For analysis, we selected clusters of shut/open intervals that appeared (by eye) to be homogeneous, with regard to P_o_. We limited the analysis to intracluster interval durations and thus excluded sojourns arising from desensitized states (shut intervals between clusters >20ms). The clusters were idealized into noise-free intervals after digitally filtering the data at 10–15 kHz (Qin, 2004). First, the idealized, intra-cluster intervals were fitted by a two-state model, C⇄O. Then, additional nonconducting and conducting states were added, one at a time, connected only to the first O state, until the log likelihood failed to improve by 10 units (Qin et al., 1997). Cluster P_O_ at each agonist concentration was calculated from the time constants of the predominant components of the shut- (τ_s_) and open-time distributions (τ_o_): τ_o_/(τ_S_+τ_o_). In this way, an equilibrium CRC was constructed as a plot of the absolute P_O_ (not normalized) versus the agonist concentration (see Figure 2).

### Equilibrium constants

Two equilibrium dissociation constants comprise efficiency, K_dC_ and K_dO_ (Figure 1; Equation 3). These, and the fully-liganded gating constant L_2_, were estimated in two ways, with both methods producing the same results.

In the primary approach, K_dC_ and K_dO_ were estimated from CRC parameters. The CRC was fitted by the Hill equation to estimate P_O_^max^ (the high concentration asymptote) and EC_50_ (the agonist concentration that produces a half-maximum P_O_). Equilibrium constants were calculated from the reaction scheme pertaining to the main activation pathway that assumes L_0_ and K_dO_ are negligible. Let x=[A]/K_dC_. For a one-site receptor the scheme is A+C⇄AC⇄AO and P_O_([A])= xL_1_/(1+x + xL_1_). For adult AChRs that have two equal and independent binding sites the scheme is A+C⇄AC⇄A_2_C⇄A_2_O (red, Figure 1B) and P_O_([A])= x^2^L_2_/(1+2x+x^2^+x^2^L_2_). Relating the two site scheme to CRC parameters, P_O_^max^ is the infinite-concentration asymptote, P_O_^min^ is the zero-concentration asymptote and EC_50_ is the concentration at which P_O_ is half P_O_^max^,

(4a) $$L_{2}=\frac{-P_{O}^{max}}{P_{O}^{max}-1}$$

(4b) $$L_{0}=\frac{-P_{O}^{min}}{P_{O}^{min}-1}$$

(4c) $$K_{dC}=\frac{{EC}_{50}\left(L_{2}+1\right)}{1+\sqrt{L_{2}+2}}$$

(4d) $$K_{dO}=\frac{K_{dC}}{\sqrt{\frac{L_{2}}{L_{0}}}}$$

where K_dO_ is solved by using Equation 1. Because L_2_=L_0_c (Equation 1), any change in L_0_ (see below) will change all three CRC parameters (P_O_^max^, P_O_^min^ and EC_50_) even if the equilibrium dissociation constant ratio K_dC_/K_dO_ remains unchanged. Knowledge of, and ability to manipulate, L_0_ was the key to measuring η.

### Voltage, L_0_ and background mutations

To reduce channel block by the agonists, the membrane was depolarized to +70 mV. To compensate for changes in τ_S_ and τ_o_ caused by depolarization, we added the background mutation εS450W. This residue is far from the binding site (M4 transmembrane region of the ε subunit), has no effect on K_dC_, and has equal but opposite effect on unliganded gating as does this extent of membrane depolarization (Jadey et al., 2011). L_0_ is 7.4x10^–7^ at V_m_=-100 mV and reduced e-fold by a 60 mV depolarization (Nayak et al., 2012). Hence, we calculate that L_0_ is 5.2x10^–7^ at V_m_=-70 mV as well as in our experiments at V_m_ = +70 mV plus εS450W.

In some conditions, for instance low efficacy agonists (Dec, TriMA, and BzTEA) and aD200A, the wt opening rate constant was small and single-channel clusters were poorly defined. Accordingly, we added background mutations to facilitate P_O_ measurements (Tables 1 and 2). These were εL269F (located in the M2 helix) and εE181W (located in strand β9) that increase the L_0_ by 179- and 5.5-fold (1084-fold for the pair) without effecting K_dC_. First, we obtained the apparent L_2_ from the CRC P_O_^max^ (Equation 4a). Second, we divided this value by the fold increase in L_0_ caused. By the background to obtain a corrected L_2_. Finally, agonist K_dC_ was estimated from EC_50_ and the corrected L_2_ (Equation 4c).

### L_0_ for αD200A

In wt adult AChRs, L_0_ is 5.2x10^–7^ at V_m_ = +70 mV (Nayak et al., 2012) and ofcourse is the same for all agonists. L_0_ has been reported previously for the mutations αK145A (Bruhova and Auerbach, 2017) and αG153S (Jadey et al., 2013). To estimate this L_0_ for αD200A, the pipette solution was free of any agonist and currents were measured at a membrane potential of −100 mV. The AChRs had added background mutations far from αD200 and each other (εL269F+εE181W+δV269A) that together increased unliganded activity substantially to allow cluster formation. Individually, these mutations increase L_0_ by 179- (Jha et al., 2009), 5.5- (Purohit et al., 2013) and 250-fold (Cymes et al., 2002), respectively (Figure 7—figure supplement 1). Assuming no interaction (Gupta et al., 2017), the expected net increase in L_0_ for this background combination is the product, ~2.5 × 10^5^. L_0_ was measured experimentally using this background plus αD200A, from the durations of intra-cluster intervals (see above). The unliganded opening (f_0_) and closing (b_0_) rate constants were estimated from the idealized interval durations by using a maximum-interval likelihood algorithm after imposing a dead time of 25 μs and L_0_ was calculated from the ratio. Using a similar approach, L_0_ was estimated previously for each mutation shown in Figure 7.

### Statistics

For single-channel CRCs, the midpoint and maximum (EC_50_ and P_O_^max^) were estimated by fitting to monophasic Hill equation (P_O_ = P_O_^max^/(1+(EC_50_/[A])*^nH^*)) using GraphPad Prism 6 (GraphPad). nH values contain information (Qin, 2010) but were not used because the number of binding sites was known a priori. A x-means cluster analysis algorithm (QUB online: qub.mandelics.com/online/xmeans.html) was used to define agonist (Figure 4A) and mutant (Figure 7A) classes, considering cluster must have at least two elements. Optimal clustering was determined based on the Sum Square Residual (SSR) and the corrected Akaike Information Criterion (AICc): for agonists SSR = 4.76 × 10^–3^ and AICc = −96.5 (n=5 classes); for mutations SSR = 2.41 × 10^–2^ and AICc=-396 (n=5 classes). Pearson’s correlation test was performed to determine correlation significance between the two variables logK_dC_ versus logK_dO_ and logL_2_ versus log[1/K_dC_]. Since the goal was to measure the correlation rather than the magnitude difference between classes, Pearson’s correlation was used instead of Cohen’s. The P-value (two-tail) and r^2^ value for that 3 agonist efficiency classes with >2 elements (Figure 5A) were 0.019 and 0.96 (*η*=0.56), <0.0001 and 0.99 (*η*=0.51) and <0.0001 and 0.99 (*η*=0.41). Significance for classes with <3 data points (*η*=0.31 and 0.46) could not be determined. The p-value (two-tail) and r^2^ value for mutants (Figure 7A) were 0.0006 and 0.83(*η*=0.56), <0.0001 and 0.98 (*η*=0.51), <0.0001 and 0.97 (*η*=0.45), <0.0001 and 0.93 (*η*=0.41), and <0.0001 and 0.98 (*η*=0.31). η is the ratio of logarithms and as such is precise.

The position of the intermediate state AC_LA_ in the catch-and-hold reaction sequence (1-η; Figure 2) is analogous to the position of the transition state (Φ orβ) in a single-step reaction. With Φthe intermediate state in the LFER is an energy barrier, whereas with η it is an energy well. Φ gives the fraction of the total energy change at ‡, and 1-η gives the fraction of the total energy change at AC_LA_.

### Agonists

Abbreviations: acetylcholine (ACh), trimethyl ammonium (TRiMA), tetramethyl ammonium, dimethylpyrrolidium (DMP), dimethylthiazolidinium (DMT), nornicotine, nicotinic (Nic), carbamylcholine (CCh), anabasine (Singh et al.), dimethylphenylpiperazinium (DMPP), benzyltrimethyl ammonium (BzTMA), choline (Cho), 3-hydroxypropyltrimethylammonium (3-OH), 4-hydroxybutyltrimethylammonium (4-OH), dimethylthiazolidinium (DMT), dimethylpyrrolidium (DMP), succinylcholine (SCh), decamethonium (Dec), epiboxidine (Ebx), epibatidine (Ebt), cytisine (Cyt), tetraethyl ammonium (TEA), tetramethyl phosphonium (TMP), varenicline (Var) and benzyltriethyl ammonium (BzTEA), Choline (Cho). Agonists were from Sigma (St. Louis, MO) except DMP, DMT, 3-OH and 4-OH that were synthesized as described previously (Bruhova et al., 2013).

## Data Availability

The source data is available in the Tables and table source data files and referred to in the legend of the associated figure.

## Additional files

MDAR checklist
