# Supplementary material for: Agonist efficiency links binding and gating in a nicotinic receptor
Source: eLife. 2023 Jul 3;12:e86496. doi: 10.7554/eLife.86496 (PMC10317499; doi:10.7554/eLife.86496)
Supplement: Table 1—source data 1. [file elife-86496-table1-data1.docx]

Table 1 – source data 1: Agonist efficiency (from literature)

| agonist | K_dC_ (mM) | K_dO_ (μM) | c | η |
| --- | --- | --- | --- | --- |
| ACh^a^ | 0.17 | 0.03 | 6707 | 0.50 |
| Nor^b^ | 0.20 | 0.04 | 4801 | 0.50 |
| CCh^a^ | 0.54 | 0.17 | 3177 | 0.52 |
| Ana^b^ | 0.23 | 0.04 | 6355 | 0.51 |
| Cho^C^ | 4.10 | 15.1 | 297 | 0.50 |
| DMPP^d^ | 0.48 | 0.13 | 3587 | 0.52 |
| 4OH^d^ | 1.20 | 1.35 | 886 | 0.50 |
| 3OH^d^ | 1.66 | 2.84 | 583 | 0.50 |
| Nic^b^ | 1.00 | 0.92 | 1084 | 0.50 |
| TMA^a^ | 1.48 | 0.62 | 2402 | 0.54 |
| DMT^a^ | 2.73 | 2.76 | 989 | 0.54 |
| DMP^a^ | 3.57 | 4.35 | 822 | 0.54 |
| Ebt^d^ | 0.03 | 0.02 | 1698 | 0.41 |
| Cyt^d^ | 0.07 | 0.1 | 650 | 0.40 |
| TEA^d^ | 1.74 | 26.77 | 65 | 0.40 |
| TMP^d^ | 0.36 | 1.47 | 244 | 0.41 |
| Var^d^ | 0.06 | 0.33 | 171 | 0.34 |
| Ebx^d^ | 0.11 | 0.05 | 2340 | 0.46 |

Low and high affinity equilibrium dissociation constants K_dC_ and K_dO_ (Fig. 2B) were calculated from CRCs (Fig. 3 and Eq. 4) after correcting L_0_ for the background (Fig. 7-figure supplement 1). c, coupling constant (Eq. 1); η, efficiency (Eq. 2). ^a^(Jadey & Auerbach, 2012), ^b^(Jadey et al., 2013), ^c^(Purohit & Grosman, 2006), ^d^(Indurthi & Auerbach, 2021).
