# Supplementary material for: Agonist efficiency links binding and gating in a nicotinic receptor
Source: eLife. 2023 Jul 3;12:e86496. doi: 10.7554/eLife.86496 (PMC10317499; doi:10.7554/eLife.86496)
Supplement: Table 2—source data 1. [file elife-86496-table2-data1.docx]

Table 2 – source data 1: Mutation efficiency

| Mutation | Ligand | K_dC_ (mM) | K_dO_  (μM) | c | η |
| --- | --- | --- | --- | --- | --- |
| αY93W^a^ | ACh | 2.01 | 1.2 | 1675 | 0.54 |
| H |  | 3.2 | 2.9 | 1103 | 0.55 |
| A |  | 1.21 | 1.3 | 931 | 0.50 |
| F |  | 2.59 | 5.8 | 447 | 0.51 |
| S |  | 6.24 | 14 | 446 | 0.55 |
| αW149Y^a^ |  | 2.41 | 3 | 803 | 0.53 |
| F |  | 12.82 | 19 | 675 | 0.60 |
| A |  | 28.83 | 260 | 111 | 0.57 |
| αY190F^a^ |  | 3.6 | 16 | 225 | 0.49 |
| W |  | 6.46 | 56 | 115 | 0.48 |
| A |  | 16.52 | 1900 | 9 | 0.35 |
| αY198F^a^ |  | 0.23 | 0.05 | 4340 | 0.50 |
| H |  | 5.7 | 9.1 | 626 | 0.55 |
| W |  | 0.61 | 0.88 | 693 | 0.47 |
| S |  | 3.9 | 12 | 325 | 0.51 |
| T |  | 9.2 | 38 | 242 | 0.54 |
| L |  | 4.1 | 21 | 195 | 0.49 |
| A |  | 7.5 | 41 | 183 | 0.52 |
| εP121L^a^ |  | 0.72 | 2.2 | 327 | 0.44 |
| Y |  | 1.27 | 3 | 423 | 0.48 |
| G |  | 1 | 1.2 | 833 | 0.49 |
| *α*G147S^b^ |  | 3.6 | 13 | 277 | 0.50 |
| A |  | 9.93 | 5.6 | 1774 | 0.62 |
| αG153^c^ | Cho | 4 | 15.39 | 260 | 0.50 |
| S |  | 0.37 | 2.03 | 182 | 0.40 |
| A |  | 0.29 | 1.71 | 168 | 0.39 |
| P |  | 0.15 | 1.02 | 151 | 0.36 |
| K |  | 0.26 | 1.76 | 146 | 0.38 |
| S | DMP | 0.18 | 0.32 | 568 | 0.42 |
| A |  | 0.19 | 0.36 | 539 | 0.42 |
| P |  | 0.2 | 0.38 | 536 | 0.43 |
| K |  | 0.23 | 0.59 | 384 | 0.41 |
| S | TMA | 0.08 | 0.07 | 1165 | 0.43 |
| A |  | 0.04 | 0.01 | 2605 | 0.44 |
| P |  | 0.1 | 0.05 | 1931 | 0.45 |
| K |  | 0.02 | 0.01 | 2116 | 0.41 |
| S | Nic | 0.09 | 0.11 | 806 | 0.42 |
| A |  | 0.11 | 0.18 | 649 | 0.42 |
| P |  | 0.04 | 0.07 | 510 | 0.38 |
| K |  | 1.2E-3 | 6.0E-5 | 1980 | 0.32 |
| E |  | 0.03 | 0.03 | 844 | 0.39 |
| R |  | 0.03 | 0.04 | 789 | 0.39 |

Low and high affinity equilibrium dissociation constants K_dC_ and K_dO_ (Fig. 2B) were calculated from CRCs (Fig. 3 and Eq. 4) after correcting L_0_ for the background (Figure 7-figure supplement 1). c, coupling constant (Eq. 1); η, efficiency (Eq. 2). ^a^(Purohit et al., 2014), ^b^(Purohit & Auerbach, 2011) , ^c^(Jadey et al., 2013)
